# Clinically-Important Brain Injury and CT Findings in Pediatric Mild Traumatic Brain Injuries: A Prospective Study in a Chinese Reference Hospital

**DOI:** 10.3390/ijerph110403493

**Published:** 2014-03-26

**Authors:** Huiping Zhu, Qi Gao, Xin Xia, Joe Xiang, Hongli Yao, Jianbo Shao

**Affiliations:** 1 School of Public Health, Capital Medical University, 10 Xitoutiao, Youanmen, Beijing 100069, China; E-Mails: zhuhuiping@ccmu.edu.cn (H.Z.); gaoqi@ccmu.edu.cn (Q.G.); 2 Beijing Municipal Key Laboratory of Clinical Epidemiology, Beijing 100069, China; 3 Hubei Provincial Center for Disease Control and Prevention, Wuhan 430079, China; E-Mail: xiaxin84@163.com; 4 Case Western Reserve University, Cleveland, OH 44106, USA; E-Mail: yxx125@case.edu; 5 Wuhan Children’s Hospital, 100 Hongkong Road, Wuhan 430016, China; E-Mail: yhl778@sina.com

**Keywords:** mild traumatic brain injury, children, emergency department, CT scan, clinically-important brain injury

## Abstract

This study investigated injury patterns and the use of computed tomography (CT) among Chinese children with mild traumatic brain injury (MTBI). We enrolled children with MTBI who were treated within 24 hours of head trauma in the emergency department of Wuhan Medical Care Center for Women and Children in Wuhan, China. Characteristics of MTBIs were analyzed by age and gender. Results of cranial CT scan and clinically-important brain injury (ciTBI) for children were obtained. The definition of ciTBI was: death from TBI, intubation for more than 24 h for TBI, neurosurgery, or hospital admission of 2 nights or more. Of 455 eligible patients with MTBI, ciTBI occurred in two, and no one underwent neurosurgical intervention. CT scans were performed for 441 TBI patients (96.9%), and abnormal findings were reported for 147 patients (33.3%, 95% CI 29.0–37.8). Falls were the leading cause of MTBI (61.5%), followed by blows (18.9%) and traffic collisions (14.1%) for children in the 0–2 group and 10–14 group. For children aged between 3 and 9, the top three causes of TBI were falls, traffic collisions and blows. Leisure activity was the most reported activity when injuries occurred for all age groups. Sleeping/resting and walking ranked in the second and third place for children between 0 and 2 years of age, and walking and riding for the other two groups. The places where the majority injuries occurred were the home for the 0–2 and 3–9 years of age groups, and school for the 10–14 years of age group. There was no statistical difference between boys and girls with regard to the activity that caused the MTBI. This study highlights the important roles that parents and school administrators in the development of preventive measures to reduce the risk of traumatic brain injury in children. Also, identifying children who had a head trauma at very low risk of clinically important TBI for whom CT might be unnecessary is a priority area of research in China.

## 1. Introduction

Traumatic brain injury (TBI) is reported to be the leading cause of death and disability in children around the world [1]. Although the TBI incidence rate was found to vary greatly between countries [2], studies from many parts of the world have showed consistently that it peaks in children and young adults. In the U.S., about 7,400 deaths, over 600,000 emergency department (ED) visits, and over 60,000 hospital admissions of children between 0 and 18 years of age were caused annually by head trauma [3,4]. Mild traumatic brain injury (MTBI), also known as cerebral concussion, is much more common than moderate or severe TBI [5,6]. Data from the U.S. Centers for Disease Control and Prevention (CDC) indicated that each year in the United States, as many as 75% of persons who had a TBI were MTBI [7]. In recent years, increasing attention has been given to the effect of TBI on children’s short- and long-term functional abilities, including memory, attention, behavior, adaptive deficits, and education [8,9,10]. Significant impairments in many domains of neuropsychological functioning have been found after TBI [11,12], including MTBI. Given the great number of MTBIs, the overall burden due to MTBIs is huge. Findings from a prospective study reported that the burden of disability resulted from TBIs is primarily accounted for by mild injuries [13], which confirmed the point that the consequences of MTBI may not be mild. 

The mechanism of TBI differs at the different developmental stages of children, and uniquely associated with children’s age [14,15]. TBIs caused by child abuse are significantly more likely to occur in children under 1 year of age (the peak incidence at 3 months) than in older age groups [16,17]. Falls are a very common reason for TBI in children between 1 and 4 years of age. For school-aged children, TBIs as results from bicycle crashes or being hit by a car while walking or riding a bicycle increase dramatically [18]. The leading external causes also differ across countries [19]. In 2006, motor vehicle collisions caused about 40%, falls caused about 20% of pediatric hospitalizations, and assault was the third leading cause in the U.S. Our recent study about characteristics and trends of hospitalized TBIs sustained by Chinese children found that falls, being struck by/striking against objects, and traffic collisions are the top external causes [15].

The primary concern for patients with a head injury is the development of a clinically-important brain injury (ciTBI) that need to be identified rapidly and treated in a timely way. Cranial computed tomography (CT) is the reference standard for the emergent diagnosis of TBI in children in the ED [20]. Over the past decades, CT use in ED has been increasing substantially in the U.S. [21]. It is estimated that CT scans are done for about 50% of children with head trauma in North American EDs [3,22]. However, less than 10% of CT scans confirm diagnosis of TBI [23]. Reduction of CT scans is of great importance for children because of the increased risk of lethal malignancies due to exposure to ionizing radiation from CT use [24,25]. In recent years, CT scan has become a common tool in EDs in China because of the greater availability of CT and their relatively low price. In some hospitals, CT scans are ordered routinely for ED patients with head trauma and are required for hospitalized patients. Research about CT use and the associated negative effects in China, however, is unavailable.

So far, pediatric traumatic brain injuries (TBIs) have not been well studied in China. No publications were found that specifically studied the children with MTBI treated at EDs. This study was conducted to address gaps in the literature on the injury patterns and use of CT for children with MTBI in China. We investigated the characteristics and the use of CT for children with MTBI treated at the ED from a large metropolitan children’s hospital in China. We sought to answer the following research questions: (1) Did the injury characteristics of MTBI cases treated in ED have unique associations with gender and age?; (2) what percentage of children with MTBI in ED received a CT scan, and what proportion of them had abnormal findings from CT scans? We also aimed to calculate the proportion of TBI cases that were clinically significant.

## 2. Methods

Our study was approved by the Institutional Review Board of Tongji Medical College, School of Public Health in Wuhan, China. Informed written consent was obtained from a legal guardian of each child involved in our study at hospital, and verbal consent for a telephone follow-up to ask about injury outcome within one month after head trauma. 

### 2.1. Setting

This study was conducted in Wuhan Medical Care Center for Women and Children (WMCCWC), which is located in Wuhan City, the capital of Hubei Province of China. WMCCWC is the largest comprehensive hospital in central China, providing a full range of clinical services, health promotion and prevention for women and children. There are four outpatient departments, two inpatient buildings and 1,112 beds in WMCCWC. Approximately 30,000 inpatients and a total of 900,000 children who come from Hubei and the neighboring provinces are treated annually at the outpatient and emergency departments (EDs) of WMCCWC. According to the data from Wuhan Bureau of Health, about 55%~60% of total pediatric outpatients and ED patients in Wuhan City are treated each year at the WMCCWC.

### 2.2. Participants

Children with traumatic brain injury treated at the ED of WMCCWC from May 1 to November 31, 2012 were enrolled in our study. Because hospitals in China are not required to collect minimum data elements and do not keep medical records for patients who are treated and discharged from the ED, a structured questionnaire was developed to collect the patients’ information, including sociodemographics, patterns of injuries, clinical history and symptoms, physical examination and CT scan findings, and TBI outcomes. If the parent or legal guardian agreed to participate in our study, a face-to-face or telephone interview was conducted with a parent or guardian of each child by a trained nurse. TBI patients with an initial Glasgow Coma Scale (GCS) score of 13 to 15 who presented to the ED of WMCCWC within 24 hours of head trauma were enrolled. We excluded children with comorbidities, or children whose CT scan was performed at another hospital without a CT scan at WMCCWC.

### 2.3. Study Measurements

#### 2.3.1. Definition of TBI

TBI cases in this study were defined using the definition of TBI of the U.S. CDC [22]: An occurrence of injury to the head with decreased level of consciousness, amnesia, and/or neurologic or neuropsychological dysfunction or diagnosis of intracranial lesion. We used U.S. CDC [7] and World Health Organization definitions [26] to define MTBI: (1) any period of transient confusion, disorientation, or impaired consciousness as recorded in the standardized questionnaire or (2) any period of amnesia around the time of injury that lasted <24 h or (3) observed signs of other neurologic or neuropsychological dysfunction and (4) Glasgow Coma Scale (GCS) score of 13 to 15 at the initial medical assessment and a GCS score of 15 at discharge from the ED. We used the modified GCS for children <3 years of age. Using the definition in the Pediatric Emergency Care Applied Research Network (PECARN) study [3], children with ciTBI were defined as death from TBI, intubation for more than 24 h for TBI, neurosurgery, or hospital admission of 2 nights or more. 

#### 2.3.2. External Cause of Injury

External causes included the following major categories: traffic collisions (with subgroups for car passengers, pedestrians, bicycle riders, electric bicycle riders, and other), falls (ground-level falls, falls from a bed, falls downstairs, and falls from a height), blows (walked or ran into a stationary object, head struck with a high-speed projectile, and others), sports, child abuse, and other. 

#### 2.3.3. Activity and Place of Injury

Activities were listed as follows: leisure activity, sport-related, walking/running, cycling, sleeping/resting, riding and other. Places where injury happened were: inside home, outside home, kindergarten/school, park/ playground, street or highway, and other.

### 2.4. Study Procedures

Information about sociodemographics, clinical history, injury characteristics, symptoms and signs was recorded by nurses before knowing the CT results. Cranial CT scans were interpreted by the pediatric neuroradiologists who did not know the clinical data. Also, investigators conducted telephone surveys of a parent or guardian of children to get patients’ outcome information within 30 days after their emergency department visits. Finally, all information was reviewed by an emergency physician to assess the CT results and ciTBI outcome.

### 2.5. Data Analysis

Categorical variables like gender, age groups and characteristics of injury in sampled children with MTBI including cause, injury place and activity at time of injury were analyzed using frequency, percentage and 95% confidence intervals (95% CI). We also analyzed the injury characteristics for children with MTBI by three age groups (0–2, 3–9, 10–14 years of age). Activity when the injury happened was compared by gender using Fisher's exact test. Percentage and the associated 95% CI of abnormal CT findings among children who had a CT scan by age were calculated. All statistical analyses were performed using the SAS statistical software. Any *p*-value less than or equal to 0.05 was considered statistically significant.

## 3. Results

In this study, a small percentage (3%) of parents or legal guardians did not agree to participate, and we finally enrolled 455 children between 0 and 14 years of age with MTBI treated in ED of the WMCCWC. The mean age of the study sample was 3.2 years (SD 3.0). The distribution by age was: 0–2 years (63.3%, 95% CI = 58.8, 67.7), 3–9 years (31.9%, 95% CI = 27.7, 36.2), and 10–14 years (4.8%, 95% CI = 3.1, 7.0). The sample included 315 boys (69.2%, 95% CI = 64.9, 73.4) and 140 girls (30.8%, 95% CI = 26.6, 35.1). Of the children included, ciTBI occurred in two, and no one underwent neurosurgical intervention. The two children with ciTBI were patient 1—a 19-month-old child ejected from the vehicle during a motor vehicle collision, who had a cerebral hemorrhage and depressed fracture of occipital bone; and patient 2—a 2-year-old child who had fallen from a third floor, sustaining multisystem trauma, intracranial contusion and hematoma. 

Of the 455 sampled children, 452 (99.3%) were discharged from the ED after simple treatment without observation, one case was discharged from the ED after observation less than 48 hours, and 2 patients were hospitalized for more than 2 days. Table 1 presents injury characteristics of the sampled children with MTBI.

**Table 1:** Characteristics of injuries in sampled 455 children with MTBI.

Characteristic	All Cases		0–2 Years		3–9 Years		10–14 Years	
	N	% (95% CI)	N	% (95% CI)	N	% (95% CI)	N	% (95% CI)
**Cause of injury**								
Falls	280	61.5(57.0–66.0)	208	72.2(66.9–77.2)	64	44.1(36.1–52.3)	8	36.4(18.0–57.1)
Fall from a bed	136	48.6(42.7–54.4)						
Ground-level falls	64	22.9(18.1–28.0)						
Fall from a height	59	21.0(16.5–26.0)						
Fall downstairs	21	7.5(4.7–10.9)						
Struck	86	18.9(15.4–22.6)	45	15.6(11.7–20.1)	33	22.8(16.3–29.9)	8	36.4(18.0–57.1)
Walked or ran into stationary object	44	51.2(40.7–61.6)						
Head struck with a high-speed projectile	25	29.1(20.0–39.1)						
Others	17	19.8(12.1–28.8)						
Traffic collisions	64	14.1(11.0–17.4)	26	9.0(6.0–12.6)	35	24.1(17.5–31.4)	3	13.6(2.8–30.7)
Pedestrian	29	45.3(33.4–57.5)						
Car passenger	22	34.4(23.3–46.4)						
Electric bicycle	8	12.5(5.6–21.6)						
Bicycle	5	7.8(2.6–15.6)						
Child abuse	19	4.2(2.5–6.2)	6	2.1(0.8–4.1)	10	6.9(3.4–11.6)	3	13.6(2.8–30.7)
Sport-related	3	0.7(0.1–1.6)	0	N.A	3	2.1(0.4–5.0)	0	N.A
Missing	2	0.4(0–1.3)	2	0.7(0.1–2.0)	0	N.A	0	N.A
Others	1	0.2(0–0.9)	1	0.3(0.0–1.4)	0	N.A	0	N.A
**Activity at time of injury**								
Leisure activity	238	52.3(47.7–56.9)	151	52.4(46.6–58.2)	78	53.8(45.6–61.8)	9	40.9(21.7–61.7)
Walking/running	89	19.6(16.0–23.3)	47	16.3(12.3–20.8)	37	25.5(18.8–32.9)	5	22.7(8.0–42.1)
Sleeping/resting	68	14.9(11.8–18.4)	59	20.5(16.0–25.3)	7	4.8(1.9–8.9)	2	9.1(0.9–24.3)
Riding	22	4.8(3.1–7.0)	8	2.8(1.2–5.0)	12	8.3(4.4–13.3)	2	9.1(0.9–24.3)
Others	19	4.2(2.5–6.2)	10	3.5(1.7–5.9)	6	4.1(1.5–8.0)	3	13.6(2.8–30.7)
Cycling	9	2.0(0.9–3.5)	7	2.4(1.0–4.5)	2	1.4(0.1–3.9)	0	N.A
Sport-related	4	0.9(0.2–1.9)	0	N.A	3	2.1(0.4–5.0)	1	4.5(0.0–17.0)
Eating	4	0.9(0.2–1.9)	4	1.4(0.4–3.1)	0	N.A	0	N.A
Missing	2	0.4(0.0–1.3)	2	0.7(0.1–2.0)	0	N.A	0	N.A
**Injury place**								
Inside home	237	51.2(46.6–55.8)	191	66.3(60.7–71.7)	44	30.3(23.1–38.1)	2	9.1(0.9–24.3)
Street or highway	70	15.4(12.2–18.8)	38	13.2(9.5–17.4)	28	19.3(13.3–26.1)	4	18.2(5.2–36.6)
Outside home	59	13.0(10.0–16.2)	37	12.8(9.2–17.0)	22	15.2(9.8–21.5)	0	N.A
Kindergarten/school	55	12.1(9.3–15.2)	5	1.7(0.6–3.6)	37	25.5(18.8–32.9)	13	59.1(38.3–78.3)
Other	23	5.1(3.2–7.3)	12	4.2(2.2–6.8)	11	7.6(3.8–12.5)	0	N.A
Park/ playground	8	1.8(0.8–3.2)	2	0.7(0.1–2.0)	3	2.1(0.4–5.0)	3	13.6(2.8–30.7)
Missing	3	0.7(0.1–1.6)	3	1.0(0.2–2.5)	0	N.A	0	N.A

The most common cause of injury was falls, accounting for 61.5% of the injuries (48.6% of which were falls from a bed, 22.9% falls from the same level, and 21.0% fall from a height). Blows (18.9%) and traffic collisions (14.1%) were the second and third frequent external causes of TBI for all children. More than half of the injury events happened inside the home and leisure activities were the most reported activity when injury occurred. When injury characteristics were analyzed by age group, falls was the leading cause of TBI, followed by blows and traffic collisions for the children between 0 and 2 years of age group, and for the 10–14 years of age group. For children aged between 3 and 9 years of age, the top three causes of TBI were falls, traffic collisions and blows. With respect to activity at time of injury, leisure activity was reported as the most common activity for all age groups. Sleeping/resting and walking were the second and third activities for children between 0 and 2 years of age, and walking and riding for the other two groups. For group 0–2 years of age, 66.3% injury events happened inside the home, and for the 3–9 years of age group, 30.3% and 25.5 injuries occurred at home or at school, respectively, but for 10–14 years of age group, 59.1% injuries happened at school, and only 9.1% happened inside the home

Table 2 shows the distribution of activity when the injury happened by gender. The most common activities that caused MTBI were the same for both boys and girls. There was no statistical significance (*p* > 0.05) between boys and girls with regard to the activity that caused the MTBI.Of 455 enrolled patients with MTBI, CT scans were performed for 441 (96.9%). The percentage of CT scan for all three age groups was 96.5%, 97.2% and 100%, respectively. Among these scans, 147 (33.3%, 95% CI 29.0–37.8) were abnormal. An abnormal scan was defined as any of the following on CT: intracranial haemorrhage or contusion, intracranial hematoma, traumatic infarction, cerebral edema, midline shift of intracranial contents, pneumocephalus, and depressed fracture of skull. The rate of abnormal CT scans for children between 10 and 14 years of age was 54.5% (95% CI 33.9–74.4). The three age groups did not differ significantly with respect to the percentage of abnormal CT scan results (see Table 3).

**Table 2:** Distribution of activity when the injury happened in children with MTBI by gender.

Activity When the Injury Happened	Male		Female	
	N	%	N	%
Leisure activity	160	51.0	78	55.7
Sport-related	2	0.6	2	1.4
Walking/running	66	20.9	23	16.4
Cycling	7	2.2	2	1.4
Sleeping/resting	46	14.6	22	15.7
Eating	1	0.3	3	2.2
Riding	17	5.4	5	3.6
Others	14	4.4	5	3.6
Missing	2	0.6	0	0.0
Total	315	100	140	100

**Table 3:** Percentage of abnormal CT findings among children who had a CT scan by age.

Age	Abnormal CT Findings /Total Scans	% (95% CI)
0~2	90/278	32.4 (27.0–38.0)
3~9	45/141	31.9 (24.5–39.8)
10~14	12/22	54.5 (33.9–74.4)
Total	147/441	33.3 (29.0–37.8)

## 4. Discussion

Previous studies about TBI in China were conducted mostly in adult populations, and very few in children. In addition, research data about treatment of Chinese children with MTBI at hospital emergency departments have not been published. This study investigated the characteristics of MTBI among Chinese children who were treated at a large children’s hospital emergency department. We found that boys were over twice as likely to be injured than girls (69.2% *vs.* 30.8% of total MTBIs cases). A similar finding from Sweden reported that boys were twice as likely as girls to sustain a head injury with symptoms of a concussion [27]. Willer *et al.* also reported that boys more likely than girls to suffer an MTBI [28]. Our result supported the finding reported by Andersson *et al.* [29] that boys and girls did not differ significantly in terms of what activities that caused a MTBI. Our finding that the most common activity when the head injury happened was playing leisure activity for both boys and girls is consistent with the previous research on what kind of activity that resulted in an MTBI [30]. When activities were analyzed separately by age, sleeping/resting and walking/running accounted for higher proportion of MTBI in children between 0 and 2 years of age. Previous study found that MTBIs are common among children less than 3 years old, and occur more often for children starting to walk or run [29], which is confirmed by our results. Agran *et al.* [31] claimed that children during the early years (children between 15 and 17 months) achieve development such as independent mobility and exploratory behavior. Children may face hazardous situation but do not have hazard awareness and avoidance skills at this special development stage, thus are susceptible to injuries [31]. Our results support these previous findings.

Our results are also consistent with previous studies that the leading cause of MTBI was falls [28,29,30], especially for younger children. These studies also reported that “fall from a height” caused the highest proportion of children MTBI. In our study, “fall from bed” was the highest percentage subgroup followed by “fall from a height”. In fact, fall from bed is a kind of fall from a height. However, given a large number of children who fell from the bed, we separated these children from the subgroup of fall from a height for analysis. Sport-related MTBIs were reported to happen increasingly with the increasing age of children in Western countries [28,30]. In our study, just three children suffered MTBI due to sport-related injury events. Until now, no publications were found that specifically address sports-related TBIs among children in China, which warrants further research on such an important health problem.

A great number of children around the world suffer from preventable injuries that happen in their homes [32]. Previous studies reported that each year, approximately 12 million nonfatal medically-treated injuries happen inside or around the home [33,34]. In our study, we found that 51.2% MTBIs happened inside the home. 

When injury place was analyzed by age, for children 0–2 years of age, most injury events (66.3%) happened inside the home. However, children between 10 and 14 years of age reported that 59.1% injuries happened at school. This finding has some implications for TBI prevention for Chinese children. Parents and school administrators need to work out preventive measures to improve the physical environment, so that children can have an opportunity to play in safer places.

Our study showed that 441 (96.9%) children with MTBI had a CT scan, and 33.3% had abnormal CT findings. This finding was consistent with previous studies reporting that CT signs of brain damage were present in one third of the mild TBI cases [35,36]. Our results indicated that the proportion of CT scans among children with TBIs is extremely high in China. Now, with health reform and increasing support in the field of primary health care by Chinese government, CT is available in every county-level hospital, even some of the health clinics in towns own a CT [37]. The cost of head CT is very cheap in China compared with other countries. All these factors lead to the overuse of CT in China [38]. Although CT scan is a great tool in the diagnosis of TBI, its disadvantages, including exposure to ionizing radiation, should be carefully considered. Previous studies found that the rate of lethal malignancies from CT is between 1 in 1,000 and 1 in 5,000 cranial CT scans for children, and the risk increases as age decreases [23,39]. Therefore, identifying children who had a head trauma at very low risk of clinically important TBI for whom CT might be unnecessary is a priority area of research for medical researchers in China. In our sample, ciTBI occurred in only two children, and no one underwent neurosurgical intervention, which supported the findings from a previous study that children with minor head trauma need neurosurgery very uncommonly [3].

Results of this study showed that children with MTBI treated in ED in this study medical center were between 0 and 14 years old. Actually, we did not limit the age interval, and our previous study based on inpatient medical records at the same medical center reported that only 1.3% (54/4230) children were between 13 and 18 years of age, and the older kids were more likely to receive medical treatment at other local hospitals (general hospital) than at a children’s hospital [15]. Now, many large general hospitals in China established critical care center to treat severe patients. Children with more severe forms of TBI are treated in these critical care centers. Patients with MTBIs are more likely treated in EDs and outpatient settings, which is why only young children with MTBI were enrolled in this study. This finding suggests that it is not a good idea to recruit TBI patients treated at EDs in China for longitudinal cohort study that aims to measure long-term disabilities and cognitive functions after TBI. It is much better to recruit TBI patients who were hospitalized in order to include more severe TBI patients. 

Our study has several limitations. First, the sample size is relatively small. Patients were only from one large children’s medical center so it should not be considered to be a random sample of MTBIs among children in China. Second, we could not calculate mild traumatic brain injury rates due to lack of denominator data. Third, selection bias might have existed because some eligible subjects were not included because parents refused to participate in our study. For these missing patients, we could not access medical records because the emergency and out-patient department of hospitals in China do not keep medical records of patients. 

## 5. Conclusions

Chinese children treated at emergency department of this large children’s hospital for mild traumatic brain injuries were mostly young children. The leading causes for the mild traumatic brain injuries were falls, traffic collisions, and blows against/by objects. Very few children with mild traumatic brain injuries had clinically-important brain injury, but almost all of them received CT scans. The wide use of CT scans in diagnosis of mild traumatic brain injuries in Chinese children and the potential long-term adverse outcome warrants additional research.
